# Knowledge of Laboratory Medicine in Medical Students: Is It Sufficient?

**DOI:** 10.30699/ijp.2020.94221.1916

**Published:** 2020-04-01

**Authors:** Hiva Saffar, Maryam Saatchi, Anahita Sadeghi, Fahimeh Asadi Amoli, Seyed Mohammad Tavangar, Farzaneh Shirani, Ali Aliasgari

**Affiliations:** 1 *Department of Pathology and Laboratory Medicine, Shariati Hospital, Tehran University of Medical Sciences, Tehran, Iran*; 2 *Department of Internal Medicine, School of Medicine, Tehran University of Medical Sciences, Tehran, Iran*; 3 *Digestive Research Institute, Tehran University of Medical Sciences, Tehran, Iran*; 4 *Department of Pathology and Laboratory Medicine, Farabi Hospital, Tehran University of Medical Sciences, Tehran, Iran*; 5 *Department of Emergency Medicine, Shariati Hospital, Tehran University of Medical Sciences, Tehran, Iran*

**Keywords:** Clinical pathology, Education, Laboratory medicine, Medical student

## Abstract

**Background & Objective:**

Appropriate use of laboratory testing is essential for achieving safe and effective care to patient. Insufficient knowledge could lead to poor case management and increase the health care costs. It is believed that education on laboratory testing for undergraduate medical student is inadequate. This study was designed to evaluate the level of knowledge of 5 and 6 year undergraduate medical students in field of laboratory medicine.

**Methods:**

Totally, 59 questions including 8 basic questions concerning individual assessment of their knowledge and 51 objective questions focusing on various stages of test ordering and interpretation were asked.

**Results:**

Thirty seven undergraduate medical students at the level of internship participated. On average, 47.9% of students evaluated themselves as “weak" in 8 self-assessment questions. There was no significant difference between responders assessment on their own knowledge in various aspects of laboratory testing (*P*=0.184). In the objective questions regarding various stages of test ordering including pre-analytic, analytic and post analytic phases,45.6%,51.9% and 50% correct answers were reported ,respectively. Comparison of the level of the knowledge of the students regarding various stages of pathology testing did not show significant difference (*P*=0.638).

**Conclusion:**

Prioritizing an effective teaching method of laboratory medicine to medical students on appropriate time should be considered in medical school curriculum for better clinical decision making and optimal modern medical care.

## Introduction

Information and objective data provided by clinical pathology laboratory plays an important role in nearly all aspects of clinical practice including diagnosis, treatment, management and prognosis (1-4). It is believed that about 60-70% of medical decisions are based on laboratory test results (2,3,5).

“Appropriate use of laboratory testing is essential for achieving safe, effective and efficient patient care”; according to a report by Centers for Disease Control and Prevention (CDC) in May 2008 (1,6). In an updated report of CDC in a May 2009 (1,7), however, it was addressed that “medical education on laboratory testing is inadequate”. It is believed that the amount of time devoted to this important subject in undergraduate medical education is not adequate (3,8).

Insufficient knowledge in this field, makes health care providers more prone to inappropriate ordering and mistakes in interpreting the results which could lead to poor case management and increase in health care costs (1,9,10).

**Table 1:** Examples of basic and objective questions focusing on knowledge of students about various stages of test ordering

Basic Question:
How do you assess your knowledge about "patient preparation and specimen collection for laboratory tests?" Good Average Weak
Objective questions:
Minimum hours of fasting required for testing of fasting blood sugar: 8 12 14 not required
Which order of sample tube collection is recommended in Lumbar Puncture? Biochemistry/Microbiology/cell count Microbiology/cell count/ Biochemistry Cell count/ biochemistry/ Microbiology
Which peripheral blood smear is representative to confirm diagnosis in a young female presented with loss of consciousness, increased serum creatinine and decreased platelet count? (presence of schistocytes, figures not included)
Which of the following tests is preferred as screening test in general population? Test with: Sensitivity:98% Specificity:60% cost per test: 10000 units Sensitivity:100% Specificity:100% cost per test: 100000 units Sensitivity:90% Specificity:90% cost per test: 10000 units

Wilson believes that the answer to the question “why so little is paid to laboratory medicine in medical school curricula?” is complex and the reasons partly vary among various schools, but slow and increment change in medical education curriculum, feeling no necessity for formal education by medical educators and limitation of resources and time to allocate to teach laboratory medicine (probably needing to overcome long-standing political opposition to substantive changes in curricula) appears three important reasons explaining why medical laboratory education is not efficient (11).

Traditionally, the major focus in pathology education has been commonly on general and systemic pathology, emphasizing on mechanisms of disease. In fact, clinical pathology/laboratory medicine has been neglected as a component of every day non pathologist physician practice (1,5,12). During the last decade, many medical schools have moved towards integration of basic science and clinical medicine and more topics are presented in integrated context (13). 

However, unfortunately, in educating laboratory medicine, clinical laboratory professionals who have implemented the tests in clinical laboratory and have the knowledge of test utility and limitations have not been involved (4).

In a large study of missed or delayed diagnosis, 35% and 37% were due to failure in ordering appropriate diagnostic tests and incorrect interpretation of test results, respectively (3,14). Analytical errors accounted only 8% of missed diagnosis (3,14).

Also data suggests that there are some shortcomings in medical students’ ability in other aspects including provider-performed microscopy (1,3,15) and pre and posttest probability in test interpretation (1,3,16).

Based on the mentioned limitations, this study was designed to evaluate the level of knowledge of 5 and 6 year undergraduate medical students in field of laboratory medicine. Also, we held a mini-course of introduction to laboratory medicine.

We hope the results could guide us to decide how to plan for more formal program in teaching clinical pathology to undergraduate medical students.

## Materials and Methods

A questionnaire was developed by a multidisciplinary team of physicians including three pathologists, Two internists and one emergency medicine specialist, all interested in medical education (the validity and reliability of the questionnaire was approved by literature review in expert panel group and test-retest method, respectively).

Totally, 59 questions including 8 basic questions concerning individual assessment of their knowledge and 51 objective questions focusing on various stages of test ordering and interpretation were asked. In fact, the first 8 questions were designed to understand how confident the students feel about the issue. Examples of basic and objective questions are summarized in Table 1. The questionnaire was designed as Multiple Choice Questions (MCQs), questions with one/two words short answers or True/False answers.

Answers were graded as correct (one positive score) or incorrect (Zero score). The target population were undergraduate medical students who had been divided into four groups. 

The plan of the study was explained and then the questionnaires were answered by the students. After that the mini-course focusing on various aspects of test ordering including pre-analysis, analysis and post analysis stages were held.

During the course, basic and theoretical concepts of clinical pathology were presented by a clinical pathologist. Test ordering and variables in pre-analytical steps such as sample collection, patient identification, storage and transport of sample or rejection criteria were explained. In analytical phase, interactive case-based discussion on peripheral blood smear exam was held. Finally, in post analytical phase, test accuracy (sensitivity, specificity and pre and post-test probability) were explained.

At the end of the course, an open question regarding to overall satisfaction of the students was asked.

The study was approved by the ethics committee of Tehran University of Medical Sciences.

## Results

Totally, 37 undergraduate medical students at the level of internship participated. The results of 8 questions focusing on self-assessment are summarized in Table 2. Five, one and two questions focusing on pre, analysis and post analysis phases were asked, respectively. 

No significant difference was observed between responders assessment on their knowledge for various aspects of laboratory testing (*P*=0.184).

The best score was devoted to the question about post analysis stage focusing on test accuracy.

In pre analytical stage, totally 1776 objective questions were asked. 810 (45.6%) and 966 (54.4%) correct and incorrect answers were recorded respectively. Among the questions, one asking about durational changes of serum cortisol was answered 100% correctly while another question asking about the minimum hours of fasting required for lipid profile testing were answered 100% incorrectly.

In analytical stage, 51.9% of answers were correct and in post analytical stage, two questions were asked where half of the answers were correct.

Comparison of the level of the knowledge of the students regarding the various stages of pathology testing, did not show significant difference (*P*=0.6).

**Table 2:** Summary of results of answers in to self-assessment questions

*** Score***	***Good(%)***	***Average(%)***	***Weak(%)***	***Total(%)***
***Test Ordering Phase***				
Pre analysis	3	42	55	100
Analysis	7.4	51.9	40.7	100
Post analysis	22.2	29.6	48.2	100

## Discussion

The number, type and complexity of laboratory testing have been increased during the last 50 years (4,11). On the other hand, there is pressure for physicians to visit more patients which interferes with the optimal time required for data collection from patient interviews and examination (4). So, nowadays health care providers rely more on laboratory tests (4,11) to make diagnosis, assess the efficacy of therapy and follow their patients (11).

In a British survey (1,17), 18-20% of medical graduates described themselves as “less than competent” in using laboratory tests.

There are some other studies which have identified shortcomings among medical students and/or non-pathology residents (1,3,14,18-20) in some aspects of laboratory medicine such as inappropriate test ordering, incorrect interpretation (3,14), microscopic exam (3,15) and calculation of pre and post-test probabilities (3,16). 

Based on our observation, participated medical students scored themselves as “weak” in about half of the questions focusing on their knowledge about various phases of pathology test ordering.

It appears that laboratory medicine education has been a neglected topic in undergraduate medical school curriculum (3). In a commentary entitled as ”insufficient teaching of laboratory medicine in US medical schools” by Laposata (21), he believes that today medical student graduates are faced with the challenge of selecting and ordering the correct test among the thousands of expensive assays on clinical laboratory menu with little or no education prior to graduation (21) . It appears that academic courses mainly had focused on “anatomic pathology” and even in revised student centered curriculums at the medical schools in United States, there are still risks of inadequate education (21). 

It is believed that dramatic growth in laboratory testing has reinforced the need for optimal formal medical student education (8).

The necessity to evaluate different education approaches for teaching laboratory medicine has been discussed (2,3,5,11,13) and the number of schools offering laboratory medicine courses has been increased since the study by Gottfried *et al.* in 1992 (8,22).

In a nearly recent survey published by Smith *et al.*, (8)they surveyed appropriate dean, department chair or undergraduate education directors at US medical schools about the state of laboratory medicine education. Among received responses, 84% offered some course works; 78% required it during the first two years and 19% also in clinical years. All required course works included a lecture component with or without laboratory session or small group problem-based learning (8). 

Some offered an elective pathology course at some parts in curriculum in addition to or in lieu of a required course which could include laboratory medicine (8).

Molinaro *et al.*, published their experience regarding the development, implementation and evaluation of a 1.5 day medical student clinical laboratory experience for the fourth year medical students. They evaluated recently acquired knowledge by post-test quiz and observed significant improvement by 4^th^ year medical students (4).

In Iran, there is limited data regarding to education of clinical pathology/laboratory medicine to medical students (5,23). Although, since 1979, the two divisions of anatomical and clinical pathology were merged and named "Asibshenasi" (24), no uniform and formal course was included regarding teaching clinical pathology (laboratory medicine) to undergraduate medical students. However, some medical schools have implemented mini-courses.

For example, as reported by Omidifar *et al.*, (5) they implemented a 1.5 working day practice course for medical students in various stages of their education. They assessed the level of knowledge of the students by pre and post-test exam. Based on their observation, students with more clinical experience achieved higher scores and the satisfaction was higher (5). Haspel *et al.*, reported their successful experience in implementation of a longitudinal integrated pathology curriculum. They believed third year is an optimal time (25).

Our observation also revealed high satisfaction rate. However; it should be mentioned that in our study, only students with high level of clinical experience were included (6^th^ year).

Some medical schools have incorporated highly successful courses concentrating on laboratory medicine in to their curriculum. For example, the University of California at San Diego, devoted 30 hours in to this subject in form of lectures which include at least 20 minutes case-based discussion(1).

Fortunately, in Iran as we know a mini course elective rotation of pathology has been included in to Tehran medical school curriculum in clinical years before internship.

The number and range of hours or year of presentation varies greatly in medical schools based on Smith report (8).

Omidifar *et al.* recommended that developing a course of clinical pathology at phases of clinical clerkship would be more beneficial (5).

Laposata (21) believes that clinical pathology should be included in medical school curriculum and in national exams, because the students only focus on the tests that they must pass to become practicing physicians (21).

An in-depth knowledge of laboratory medicine is vital for all graduated physicians (1). On the other hand, overloaded curriculum limits devoting specific time to this subject (5).

Our findings revealed inadequate knowledge of clinical stage medical students about various phases of pathology testing. Although, we observed a high satisfaction rate after the mini-course of laboratory education program, we did not assess the knowledge of students by post-test or their performance which was one of our limitations.

We believe that the necessity of developing and implanting of theoretical or practical courses is inevitable. Moreover, the trends in carrier choice has been studied (26) and in this regard, like others we think the positive perception of pathology among medical students should be enhanced and the profile of pathology should be raised to attract future graduates to adopt pathology as a career choice (27,28).

## Conclusion

Finally, although medical schools are still ongoing to establish the most effective teaching method of clinical pathology/laboratory medicine to medical students, there is no doubt in prioritizing education of this subject. The positive impact on better clinical decision making and modern medical care would be inevitable.
